# Comparative Cytogenetics of Lace Bugs (Tingidae, Heteroptera): New Data and a Brief Overview

**DOI:** 10.3390/insects13070608

**Published:** 2022-07-06

**Authors:** Natalia V. Golub, Viktor B. Golub, Boris A. Anokhin, Valentina G. Kuznetsova

**Affiliations:** 1 Department of Karyosystematics, Zoological Institute, Russian Academy of Sciences, Universitetskaya emb.1, St. Petersburg 199034, Russia; cnidaria@yandex.ru (B.A.A.); valentina.kuznetsova@zin.ru (V.G.K.); 2 Department of Zoology and Parasitology, Voronezh State University, Universitetskaya sq.1, Voronezh 394006, Russia; v.golub@inbox.ru

**Keywords:** karyotype, chromosome number, sex chromosomes, FISH, 18S rDNA, (TTAGG)_n_, Tingini, Acalyptaini, Tinginae

## Abstract

**Simple Summary:**

Tingidae, or lace bugs, is a family of herbivorous true bugs, with approximately 2600 identified species in 318 genera classified in two or sometimes three subfamilies, among which the largest subfamily Tinginae comprises about 2500 species. Here, an account is given of the karyotypes of 16 lace bug species studied using conventional chromosomal techniques and FISH with two repetitive DNA probes, 18S rDNA and (TTAGG)_n_. We also summarize and analyze all information accumulated to date on karyotypes of lace bugs. In general, such information is available for 60 species and 22 genera of Tinginae. We show that lace bugs are characterized by: (1) very conservative karyotypes, with six pairs of autosomes in a haploid set; (2) either an XY or X(0) sex chromosome system; (3) a conventional sequence of sex chromosome divisions in male meiosis; (4) absence of the (TTAGG)_n_ mechanism for maintaining telomere integrity; (5) four discrete patterns of 18S rDNA chromosomal localization. We conclude that the search for chromosomal landmarks is of paramount importance for the characterization of the lace bug cytogenetics in more detail.

**Abstract:**

The lace bug family Tingidae comprises more than 2600 described species in 318 genera that are classified into the subfamilies Tinginae (about 2500 species and 300 genera), Cantacaderinae, and Vianadinae. We provide data on karyotypes of 16 species belonging to 10 genera of the tribes Tingini and Acalyptaini (Tinginae) studied using conventional chromosome staining and FISH. The species of Tingini possess 2n = 12A + XY, whereas those of Acalyptaini have 2n = 12A + X(0). FISH for 18S rDNA revealed hybridization signals on one of the medium-sized bivalents in species of both tribes. FISH with a telomeric probe TTAGG produced no signals in any species. In addition, we provide a list of all data obtained to date on Tingidae karyotypes, which includes 60 species from 22 genera of Tinginae. The subfamily is highly conservative in relation to the number and size of autosomes, whereas it shows diversity in the number and chromosomal distribution of the rDNA arrays, which may be located either on a pair of autosomes (the predominant and supposedly ancestral pattern), on one or both sex chromosomes, or on an autosome pair and the X. The absence of the “insect” telomeric sequence TTAGG in all species implies that Tinginae have some other, yet unknown, telomere organization.

## 1. Introduction

Tingidae, or lace bugs, is a large worldwide family of herbivorous true bugs (Heteroptera) found in all major zoogeographic regions, with most species represented in tropics and subtropics. These tiny insects have a mean length of 4 mm and live on herbaceous plants, on the roots and in the canopies of trees, and at ground level on mosses. Most of the species live and feed on a single or on a group of host species [1]. Tingidae are part of the superfamily Tingoidea [2,3,4,5] or Miroidea [6,7,8] and belong to the largest true bug infraorder Cimicomorpha [7,8]. Based on morphological characters, paleontological data and molecular sequence data analysis, Tingidae are the most closely related to the largest true bug family Miridae [9,10]. Tingidae comprise more than 2600 described species in 318 genera and three subfamilies: Tinginae (approximately 2500 species) with tribes Phatnomini, Litadeini, Ypsotingini, Tingini, and Acalyptaini; Cantacaderinae with tribes Cantacaderini, Ceratocaderini, and Carldrakeanini; and Vianaidinae [8,11,12,13,14].

Lace bugs are characterized by holokinetic (=holocentric) chromosomes like all other Heteroptera. The karyotypes of Tingidae have beens described and discussed in many publications and reviews [15,16,17,18,19,20,21,22,23,24,25], including in a series of our recent papers [14,26,27,28,29]. Ueshima [23] published a monograph on the heteropteran karyotypes, in which he cited, among other aspects, the karyotypes of 16 lace bug species studied until that time. To date, the karyotypes of 47 species (approximately 1.8% of the described species) have been studied. All these species belong to the largest subfamily Tinginae, to tribes Tingini (with more than 2300 species described) and Acalyptaini (with about 180 species). Most species were examined using conventional cytogenetic techniques that allow information to be obtained on the number of chromosomes, chromosomal mechanisms of sex determination, and behavior of the chromosomes in meiosis. In particular, the lace bugs were shown to have an orthodox sequence of sex chromosome divisions in male meiosis (in contrast to the inverted sequence characteristic of the great majority of true bugs in which sex chromosomes divide equationally at the first anaphase and reductionally at the second anaphase [23]). It was also shown that the lace bugs display very conservative karyotypes, with 12 autosomes found in all species studied so far and the XY as the most typical sex chromosome system [14,23,26,27,28,29]. Grozeva and Nokkala [25] used C-banding for the first time to study the distribution of constitutive heterochromatin in the karyotypes of lace bug species. After studying 13 species sharing the same karyotype of 2n = 12 + XY, they showed that C-heterochromatin is more often localized in telomeres, but in some species it nevertheless localizes in interstitial positions evidencing that a quite substantial redistribution of chromosome material within chromosomes might occur without fragmentations or fusions. In our studies, we applied fluorescence in situ hybridization (FISH) with 18S rDNA- and TTAGG- telomeric probes for the first time. We have shown that lace bugs, firstly, have various locations of the major ribosomal genes, with a pronounced tendency toward the autosomal location, and, secondly, they do not have the “insect” telomere motif (TTAGG)_n_ [26,28,29].

In the development of these studies, we aimed in the present study to analyze the karyotypes of 16 species of lace bugs belonging to the subfamily Tinginae. All but three of these species were studied using FISH with 18S rDNA and “insect” telomeric TTAGG probes. Finally, we present an updated list of the lace bug species studied so far in terms of karyotypes and summarize all known cytogenetic information on Tingidae for better presentation of the patterns of chromosomal evolution in the family.

## 2. Materials and Methods

Insects were collected mainly in the mountains and foothills of the North Caucasus, Transcaucasia, Crimea, and Altai, as well as in the East European forest steppe and steppe of the European part of Russia. The species studied, the localities, and the quantity of the material are listed in Table 1.

Only adult males were analyzed. Specimens were fixed immediately after collection in the field in 3:1 (ethanol/acetic acid) fixative and stored in the same fresh fixative in the laboratory at 4 °C until slides were made. Testes were dissected out in a drop of 45% acetic acid on the slide, covered with a coverslip and squashed by gently pressing. The coverslip was removed with a razor blade after freezing with dry ice, and the slide was then dehydrated in fresh fixative (3:1) and air dried. Prior to staining, the preparations were examined using phase contrast microscopy. In order to study the standard karyotypes, the preparations were stained according to a Schiff–Giemsa method developed by Grozeva and Nokkala [30]. In order to induce a good contrast with low background, we empirically extended staining in Schiff’s reagent to 20 min and subsequent Giemsa staining in Sorensen’s buffer, pH 6.8, to 20 min. For each species, at least 30 meiotic metaphases were examined to determine the chromosome number, the sex chromosome system, and the structure of the karyotype.

To determine the distribution of the major rDNA, i.e., tandemly repeated arrays of genes constituting nucleolus organizing regions (NORs), and to determine whether telomeres are composed of the “insect” telomere (TTAGG)_n_ motif, FISH with 18S rDNA- and TTAGG- probes was carried out according to a protocol [31]. Briefly, the target 18S rDNA probe (about 1200 bp fragment) was amplified via polymerase chain reaction (PCR) with no template control (NTC) and labeled with biotin-11-dUTP (Fermentas, EU) using specific primers: 18S_F (5′-GATCCTGCCAGTAGTCATATG-3′) and 18S_R (5′-GAGTCAAATTAAGCCGCAGG-3′) [32]. Genomic DNA was extracted from the true bug *Pyrrhocoris apterus* (Linnaeus, 1758). An initial denaturation period of 3 min at 94 °C was followed by 35 cycles of 30 s at 94 °C, annealing for 30 s at 55 °C, and extension for 110 s at 72 °C, with a final extension step of 3 min at 72 °C. The telomeric probe TTAGG was amplified by PCR and labeled with rhodamine-5-dUTP (GeneCraft, Köln, Germany) using primers: TTAGG_F (5′-TAACCTAACCTAACCTAACCTAA-3′) and TTAGG_R (5′-GGTTAGGTTAGGTTAGGTTAGG-3′) [33]. An initial denaturation period of 3 min at 94 °C was followed by 30 cycles of 45 s at 94 °C, annealing for 30 s at 50 °C, and extension for 50 s at 72 °C, with a final extension step of 3 min at 72 °C. The chromosome preparations were treated with 100 μg/mL RNAse A and 5 mg/mL pepsin solution to remove excess RNA and proteins. Chromosomes were denatured in the hybridization mixture containing labeled 18S rDNA- and TTAGG- probes with an addition of salmon sperm blocking reagent and then hybridized for 42 h at 37 °C. The 18S rDNA probe was detected with NeutrAvidin-Fluorescein conjugate (Invitrogen, Karlsbad, CA, USA). The chromosomes were mounted in an antifade medium (ProLong Gold antifade reagent with DAPI, Invitrogen) and covered with a glass coverslip.

As a positive control for the TTAGG telomeric probe, a barklouse (Psocomorpha) species *Psococerastis gibbosa* (Sulzer, 1766), which is proven to be TTAGG-positive [34], was used.

Observations and image capture were carried out using a Leica DM 6000 B microscope with a 100×objective, Leica DFC 345 FX camera, and Leica Application Suite 3.7 software with an Image Overlay module (Leica Microsystems, Wetzlar, Germany). The filter sets applied were A, L5, and N21 (Leica Microsystems). At least five good-quality early to late metaphase cells from each male were used for analyzing hybridization signals. The specimens, from which the chromosome preparations were obtained, are stored at the Zoological Institute RAS (St Petersburg, Russia).

## 3. Results

### 3.1. Tribe Tingini

**Figure 1:**
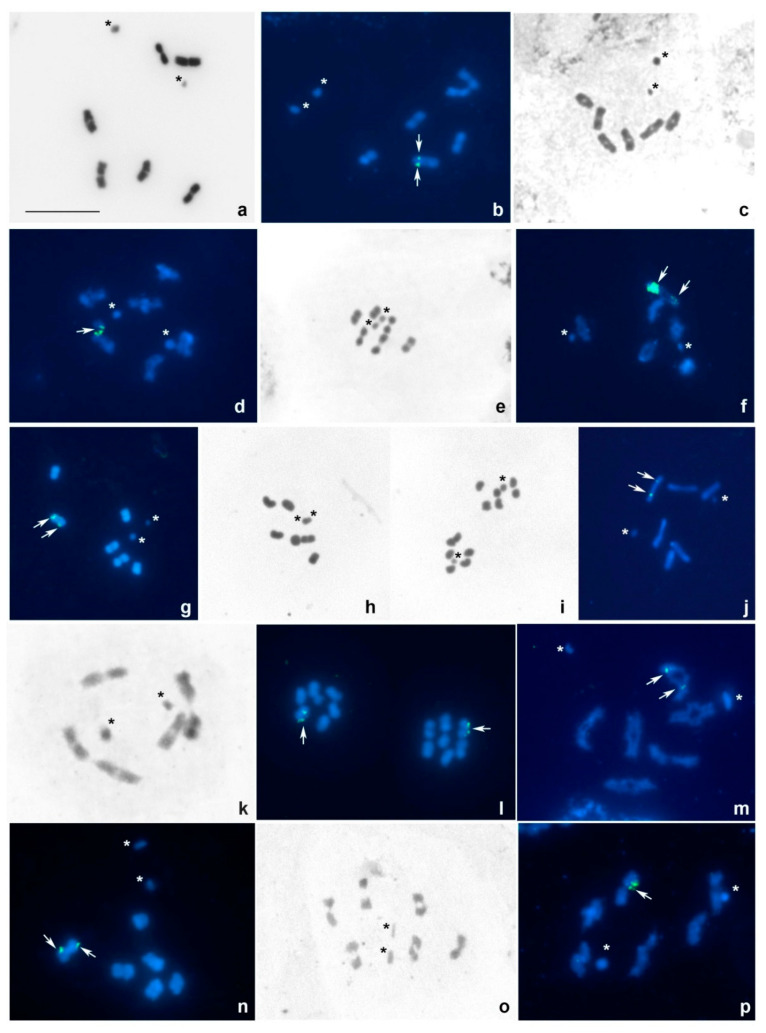
(**a**–**p**). Meiotic karyotypes of Tingini species after conventional staining and double FISH using 18S rDNA and telomeric TTAGG as probes. (**a**) *Agramma blandulum*, MI; (**b**) *Agramma minutum*, MI; (**c**) *Copium adumbratum*, MI; (**d**,**e**) *Copium brevicorne,* MI (**d**), diakinesis (**e**); (**f**) *Copium clavicorne*, diakinesis; (**g**–**i**) *Corythucha arcuata,* MI (**g**,**h**), MII (**i**); (**j**) *Corythucha ciliata,* MI; (**k**,**l**) *Galeatus affinis,* MI (**k**), MII (**l**); (**m**) *Physatocheila* *putshkovi*, diakinesis; (**n**) *Physatocheila* *smreczynskii*, MI; (**o**) *Stephanitis oschanini*, MI; (**p**) *Tingis brevicornis,* early metaphase I. Sex chromosomes are marked by asterisks; 18S rDNA signals are shown by arrows; TTAGG signals are absent. Bar = 10 mkm.

*Agramma blandulum* and *A. minutum*, 2n = 14, XY (Figure 1a,b).

In males of both species, six bivalents of autosomes and two sex chromosome univalents, X and Y, were observed at spermatocyte metaphase I (MI), assuming they have the same diploid number, 2n = 14, and a similar sex determination system of the XY type (meioformula: 2n = 6AA + XY). All bivalents decrease in size more or less linearly. Sex chromosomes appear different in size in *A. blandulum* (Figure 1a) while similar in size in *A. minutum* (Figure 1b) being the smallest elements of the karyotype in both species. At MI, they are located at a distance from each other suggesting them to be non-chiasmatic, and they regularly co-orientate at this stage (Figure 1a,b).

Only *A. minutum* was studied using FISH. Fluorescent signals of the 18S rDNA probe were detected in an interstitial position on one homologue of a medium-sized bivalent; no signals of the TTAGG telomeric probe were detected (Figure 1b).

*Copium adumbratum*, *C. brevicorne*, and *C. clavicorne*, 2n = 14, XY (Figure 1c–f).

In males of all three species, six bivalents of autosomes and two sex chromosome univalents, X and Y, were observed at different stages of the first meiosis, including diakinesis and MI (Figure 1c–f). Species, therefore, have 2n = 14 and an XY sex determination system (meioformula: 2n = 6AA + XY). The bivalents are of similar size. Sex chromosomes are non-chiasmatic, while clearly co-oriented (Figure 1c–e), and represent the smallest elements of the karyotype. In *C. brevicorne* (Figure 1d,e) and *C. clavicorne* (Figure 1f), sex chromosomes are slightly different in size, while in *C. adumbratum* (Figure 1c), one of the sex chromosomes, is significantly larger than the other. *C. clavicorne* was previously studied by Grozeva and Nokkala [25] who described the same karyotype for males collected from Teucrium chamaedrys Linnaeus, 1753, in Bulgaria.

Only *C. brevicorne* and *C. clavicorne* were studied using FISH. Fluorescent signals of the 18S rDNA probe were detected in an interstitial position on one homologue of a medium-sized bivalent in the first species (Figure 1d) and on both homologues of a medium-sized bivalent in the second species (Figure 1f). No signals of the TTAGG telomeric probe were detected in both species (Figure 1d,f).

*Corythucha arcuata* and *C. ciliata*, 2n = 14, XY (Figure 1g–j).

In males of both species, six bivalents of autosomes and two sex chromosome univalents, X and Y, were observed at MI, assuming they have 2n = 14 and an XY sex determination system (meioformula: 2n = 6AA + XY). All bivalents are of similar size. Sex chromosomes are the smallest elements of the karyotype, and one of them is definitely larger than the other. They are located at a distance from each other being non-chiasmatic and clearly co-orientated (Figure 1g,j) or form a pseudo-bivalent at MI (Figure 1h). Two sister metaphase II (MII) nuclei, each with six autosomes and an X- or Y- sex chromosome, respectively, were observed, confirming thus that the first meiotic division was reductional for the sex chromosomes. Each MII plate tends to be radial, with the sex chromosome lying in its center (Figure 1i). *C ciliata* was previously studied by Grozeva and Nokkala [25] who described the same karyotype for males collected from *Platanus acerifolia* Willdenow, 1805, in Bulgaria.

In each of the species, fluorescent signals of the 18S rDNA probe could be seen on both homologues of a medium-sized bivalent in a sub-terminal position in *C. arcuata* and in an interstitial position in *C. ciliata*; in both species, the signals were more pronounced in one homologue of the bivalent. No signals of the TTAGG telomeric probe were detected (Figure 1g,j).

*Galeatus affinis*, 2n = 14, XY (Figure 1k,l).

In males, six bivalents of autosomes and two sex chromosome univalents, X and Y, were observed at different stages of the first meiosis, assuming they have 2n = 14 and an XY sex determination system (meioformula: 2n = 6AA + XY). At early MI presented in Figure 1k, all bivalents are of similar size. Sex chromosomes are the smallest elements of the karyotype, and one is slightly larger than the other chromosome. They are placed at a sufficient distance from each other being clearly co-oriented at this stage.

Figure 1l shows two sister MII nuclei, each with six autosomes and an X- or Y- sex chromosome, respectively, confirming thus that the first meiotic division was reductional for the sex chromosomes. Each MII plate is radial with the sex chromosome lying in its center. Fluorescent signals of the 18S rDNA probe could be seen in a sub-terminal position on each chromatid of an autosome; no signals of the TTAGG telomeric probe were detected (Figure 1l).

*Physatocheilaputshkovi* and *Ph.* *smreczynskii*, 2n = 14, XY (Figure 1m,n).

In males of both species, six bivalents of autosomes and two sex chromosome univalents, X and Y, were observed at different stages of the first meiosis, including diakinesis and MI (Figure 1m,n) assuming they have 2n = 14 and an XY sex determination system (meioformula: 2n = 6AA + XY). All bivalents are of similar size. Sex chromosomes are the smallest elements of the karyotype, and one of them is definitely larger than the other chromosome. Sex chromosomes are placed at a distance from each other being non-chiasmatic and clearly co-oriented at these stages. *Ph.* *smreczynskii* was previously studied by Grozeva and Nokkala [25] who reported the same karyotype for specimens collected from *Prunus padus* Linnaeus, 1753, and *Sorbus* sp. in Bulgaria.

In each of the species, fluorescent signals of the 18S rDNA probe could be seen in an interstitial position on both homologues of a medium-sized bivalent; no signals of the TTAGG telomeric probe were detected (Figure 1 m,n).

*Stephanitis oschanini*, 2n = 14, XY (Figure 1o).

In males, six bivalents of autosomes and two sex chromosome univalents, X and Y, were observed, assuming they have 2n = 14 and an XY sex determination system (meioformula: 2n = 6AA + XY). All bivalents are of similar size. Sex chromosomes are co-oriented and represent the smallest elements of the karyotype, and one of them is clearly larger than the other (Figure 1o).

The karyotype was not studied using FISH.

*Tingis brevicornis*, 2n = 14, XY (Figure 1p).

In males, six bivalents of autosomes and two sex chromosome univalents, X and Y, were observed at different stages of the first meiosis, assuming they have 2n = 14 and an XY sex determination system (meioformula: 2n = 6AA + XY). At early MI presented in Figure 1p, all bivalents are of similar size; sex chromosomes are the smallest elements of the karyotype, and one is slightly larger than the other chromosome; they are placed at a sufficient distance from each other suggesting them to be non-chiasmatic.

Fluorescent signals of the 18S rDNA probe could be seen in an interstitial position on both homologues of a medium-sized bivalent; no signals of the TTAGG telomeric probe were detected (Figure 1p).

### 3.2. Tribe Acalyptaini

**Figure 2:**
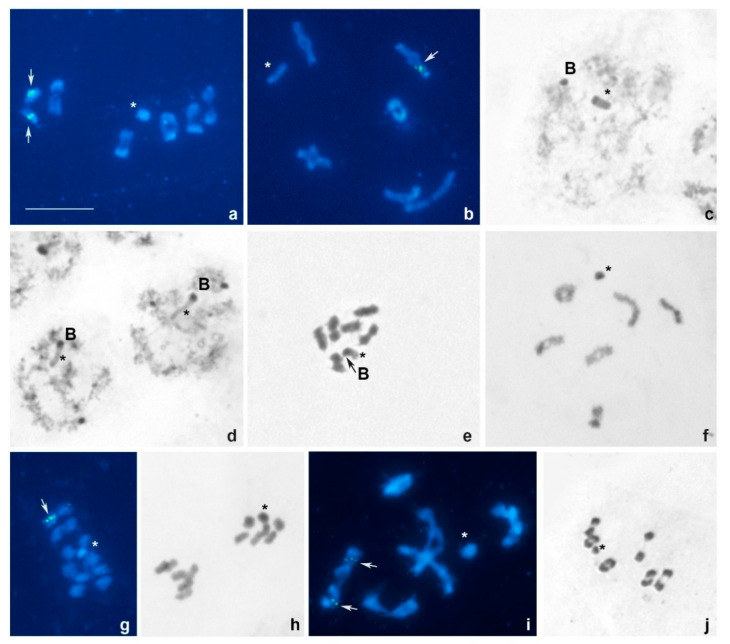
(**a**–**j**). Meiotic karyotypes of Acalyptaini species after conventional staining and double FISH using 18S rDNA and telomeric (TTAGG)_n_ as probes. (**a**) *Acalypta gracilis*, MI; (**b**–**e**) *Acalypta hellenica*, diakinesis (**b**), diffuse stage (**c**), diplotene (**d**), MI (**e**); (**f**–**h**) *Derephysia* (*Paraderephysia*) *cristata*, diakinesis (**f**), MI (**g**), MII (**h**); (**i**,**j**) *Kalama beckeri*, MI (**i**), diakinesis (**j**). Sex chromosomes are marked by asterisks; B—B-chromosome; 18S rDNA-FISH signals are shown by arrows; TTAGG signals are absent. Bar = 10 mkm.

*Acalypta gracilis* and *A. hellenica*, 2n = 13, X(0) (Figure 2a–e).

In males of both species, six bivalents of autosomes and one univalent chromosome, the X, were observed at different stages of the first meiosis assuming that they have 2n = 13 and an X(0) sex determination system (meioformula: 2n = 6AA + X). Bivalents are fairly differentiated with respect to their size; however, it is difficult to divide them objectively into size groups because their sizes decrease more or less linearly. The sex chromosome is quite large, being close in size to one of the large half-bivalents (Figure 2a,b). In *A. hellenica*, one of the two males analyzed carried an extra or B-chromosome in every cell suggesting it to be mitotically stable. During the diffuse stage, the autosomes underwent de-condensation while X- and B- chromosomes remained condensed and heteropicnotic. At this stage, X and B appeared well separated from each other (Figure 2c), although at diplotene they typically formed a pseudo-bivalent (Figure 2d). At MI, these chromosomes also appeared co-orientated, with the B being heteropicnotic and the X negatively heteropicnotic (Figure 2e).

Fluorescent signals of the 18S rDNA probe could be seen in an interstitial position on both homologues of a medium-sized bivalent in *A. gracilis*, but on one homologue of a medium-sized bivalent in *A. hellenica*, no signals of the TTAGG telomeric probe were detected (Figure 2a,b).

*Derephysia* (*Paraderephysia*) *cristata*, 2n = 13, X(0) (Figure 2f–h).

In males, six bivalents of autosomes and one univalent chromosome, the X, were observed at different stages of the first meiosis assuming that they have 2n = 13 and an X(0) sex determination system (meioformula: 2n = 6AA + X) (Figure 2f,g). At MI, autosomal bivalents show the axial orientation on the spindle, and the univalent X lies at the equator (Figure 2g). Bivalents decrease in size more or less linearly. Figure 1h shows two sister MII nuclei, with six autosomes plus the X and with six autosomes only, respectively. This confirms that the first meiotic division was reductional for both the autosomes and sex chromosome.

Fluorescent signals of the 18S rDNA probe could be seen in an interstitial position on one homologue of a medium-sized bivalent; no signals of the TTAGG telomeric probe were detected (Figure 2g).

*Kalama beckeri*, 2n = 13, X(0) (Figure 2i,j).

In males, six bivalents of autosomes and one univalent chromosome, the X, were observed at different stages of the first meiosis assuming that they have 2n = 13 and an X(0) sex determination system (meioformula: 2n = 6AA + X). Bivalents decrease in size more or less linearly (Figure 2i,j).

Fluorescent signals of the 18S rDNA probe could be seen in an interstitial position on both homologues of a medium-sized bivalent; no signals of the TTAGG telomeric probe were detected (Figure 2i).

## 4. Discussion

### 4.1. Chromosome Numbers and Sex Chromosome Systems

In general, we karyotyped here 16 species belonging to 10 genera of the largest lace bug subfamily Tinginae. Of these, nine species belonging to six genera of the tribe Tingini were found to have 2n = 12 + XY, whereas four species belonging to three genera of the tribe Acalyptaini 2n = 12 + X(0). All but three species were karyotyped for the first time, while the karyotypes, we revealed in *Copium clavicorne*, *Corythucha ciliata*, and *Physatocheila smreczynskii* (Tingini), were the same as previously reported [25]. The cytogenetic characteristics observed in 16 species are generally in agreement with those already described for all representatives of the family Tingidae [15,16,17,18,19,20,21,22,23,24,25,26,27,28,29].

Taking into account our new data, the karyotypes of 60 lace bug species (about 2.3% of the described ones) belonging to 22 genera (about 8.1% of the accepted ones) are known (Table 2).

Among the heteropterans cytogenetically studied, lace bugs demonstrate exceptional karyotypic conservatism, which is manifested primarily in the number of autosomes. It must be emphasized however that the currently available cytogenetic information refers only to the largest subfamily Tinginae. In the lace bug male karyotypes, 12 autosomes are invariably present, although the sex chromosome system may be either X(0) or XY, which was also confirmed in our present study. To be precise, 2n = 10 + XY was reported for *Acalypta parvula* from the British Isles [18], but this report is questionable, since 2n = 12 + X(0) was found in males of this species collected in Finland, being confirmed with convincing karyotype photos [25].

Most of the studied lace bugs (48 species from 18 genera, i.e., about 80% of both) have the XY sex chromosome system, and none of these genera contain species with X(0). The latter system appears to be characteristic of the genera *Acalypta* Westwood, 1840; *Derephysia* Spinola, 1837; *Kalama* Puton, 1876; and *Dictyonota* Curtis, 1827. In these four genera, the X(0) is found in all but one (see below) species, including four ones explored in the present paper, assuming that these genera may form a separate phylogenetic lineage within Tinginae. Quite recently, based on a number of significant morphological characteristics and on the distinctive sex chromosome pattern, the tribe Acalyptini Blatchley, 1926, was resurrected with a changed name Acalyptaini for these closely related and almost exclusively Holarctic genera [14].

It should nevertheless be noted that only about 6.7% of species have been studied cytogenetically in the Acalyptaini (12 out of approximately 180 described species), so further research may yet give unexpected results. In fact, one such result is already known. Southwood and Leston [18] reported an XY system for *Dictyonota fuliginosa*; however, they did not provide any photo or drawing of the karyotype, and this result therefore needs to be verified. Note that the second species of this genus, *D. strichnocera*, was shown to have the X(0) system [29].

Both the data obtained in the present study and those published earlier [23,25,26,27,28,29] suggest that lace bugs are conservative not only with respect to the number of autosomes but also in respect to the structure of the karyotype. In each male meiotic karyotype, the size of bivalents gradually decreases, preventing size groups from being distinguished or individual chromosomes from being identified. There is unfortunately a problem in lace bugs with obtaining mitotic cells (where chromosome size can be accurately identified), and only a few cases have been described. However, the analysis of chromosomes in some spermatogonial and oogonial nuclei of several species confirmed the above pattern [24,25]. In addition, the chromosomes of lace bugs, like those of true bugs in general [23], are holokinetic and lack morphological markers (the centromeres) that makes the identification of individual chromosomes in a karyotype even more difficult.

Both a stable number of autosomes and a stable karyotype structure suggest that large chromosomal rearrangements, such as fusion and fissions, did not play a significant role in the karyotype evolution and species diversification of lace bugs, at least if we mean the subfamily Tinginae. However, in their evolution there were replacements of the sex chromosome systems. The predominant XY system in Tingidae is known to also be the most common system in Cimicomorpha [35] and in Heteroptera as a whole [36]. The question of which system, XY or X(0), was evolutionarily initial in Heteroptera has been actively debated in the literature for some time, and various arguments were given in support of both opinions [23,24,25,36]. Some of the arguments presented, e.g., the discovery of the XY system in some “primitive” taxa [24,37], support the hypothesis that XY was the ancestral character state in Heteroptera. Among lace bugs, the very first X(0) species, *Kalama tricornis*, was discovered by Nokkala and Nokkala [24], and they hypothesized that this system resulted from the loss of the Y chromosome in the process of evolution. At present, when there are more data, this hypothesis finds further support (see [29]). Moreover, based on the available data, it can be assumed that the loss of the Y could have occurred in the closest ancestor of the tribe Acalyptaini [14] confirming thus that it is a more recent tribe in relation to the tribe Tingini.

However, a new XY system can theoretically evolve independently many times in the Acalyptaini displaying the X(0) as the ancestral system. According to [38,39], the Y-chromosome can originate from a pre-existing mitotically stable B-chromosome, which is first incorporated into an achiasmatic segregation mechanism with the X-chromosome in male meiosis and then becomes fixed in the karyotype as a Y-chromosome. The latter, however, carries no male determining genes [39]. Such an origin could potentially account for the abovementioned situation in *Dictyonota fuliginosa* that, according to [18], has 12 autosomes in the diploid complement, like all Acalyptaini, while the XY system, B-chromosomes were previously described in *Acalypta parvula* [25] and are found in our present study in *A.* *hellenica.* Unfortunately, in both cases, their behavior in meiosis could be traced back only to MI. In *A.* *hellenica*, X- and B-chromosomes typically appeared as univalents during diffuse stage, as a pseudo-bivalent at diplotene and as a co-oriented pair at MI (the so-called “touch and go pairing”). Based on this observation, it seems likely that these chromosomes will undergo regular segregation at anaphase I (AI).

### 4.2. Male Meiosis

In the present study, we were able to trace in some detail the behavior of chromosomes in the XY species *Corythucha arcuata*, *Galeatus affinis*, and *Copium brevicorne* and in the X(0) species *Derephysia cristata*. In all these species, both autosomes and sex chromosomes were observed to segregate reductionally during the first meiotic division, suggesting that they will therefore separate equationally during the second division (although, unfortunately, neither anaphase II nor telophase II was present in the studied males). This result was quite expected. In the vast majority of eukaryotic organisms, all the chromosomes are known to reduce in number during the first division (therefore called “reductional division”), whereas the chromatids separate during the second division (“equational division”) of male meiosis, and this pattern is known as a “pre-reduction” [40]. Hemiptera in general share this orthodox pattern; however, in true bugs, sex chromosomes typically undergo “post-reduction”, i.e., they divide equationally at AI and separate reductionally at AII, the autosomes remaining their conventional sequence of divisions [23]. The phenomenon is called “inverted sequence of sex chromosome divisions”. Although examples of sex chromosome pre-reduction occur sporadically in certain families (see, e.g., [41,42,43,44,45,46]), lace bugs represent the only heteropteran family showing the conventional pre-reductional meiotic pattern in all species studied so far in this respect ([23,25,26], present study).

### 4.3. Patterns of 18S rDNA Localization

Such features of lace bugs as holokinetic chromosomes, the extraordinary stability of the number of autosomes, and similar sizes of chromosomes in karyotypes of different species make it difficult to conduct research on the comparative cytogenetics of the family Tingidae.

In most eukaryotic genomes, rDNAs are organized into two distinct multigene families, one coding for 5S rRNA and the other coding for 45S rRNA. The 45S rDNAs (28S, 18S, and 5.8S) are highly repeated and arranged in tandem in one or a few loci situated on one or several chromosomes. Characterization of the number and distribution of rDNA arrays (loci) in the karyotype using FISH provides chromosomal landmarks useful for phylogenetic and evolutionary studies.

In order to search for chromosomal markers and to characterize the lace bug cytogenetics in more detail, we used FISH with 18S rDNA and “insect” telomeric TTAGG probes ([26,27,28,29], present paper). After a single publication dedicated to C-banding in chromosomes of several species [25], our FISH-based studies mark the first steps towards the identification of individual chromosomes in lace bug karyotypes.

In the present work, we added new data by studying the location of 18S rDNA loci in the karyotypes of 13 species belonging to 10 genera of the tribes Tingini and Acalyptaini. Despite quite wide taxonomic sampling, all the species appeared similar having rDNA clusters on a pair of medium-sized autosomes. However, if we analyze all the data available to date, we can see that the distribution of rDNA in lace bug karyotypes is not so uniform. Data are currently available for 38 species from 16 genera of the subfamily Tinginae, tribes Tingini and Acalyptaini (Table 2). In general, four patterns of rDNA localization are found in the lace bug species. These patterns are: (i) two rDNA sites (on one autosomal bivalent, “AA pattern”); (ii) one rDNA site (on the X-chromosome, “X pattern”); (iii) two rDNA sites (on X and Y respectively, “X + Y pattern”); (iv) three rDNA sites (on one autosomal bivalent and on the X-chromosome, “AA + X pattern”). The same four location patterns are the main ones recognized in another cimicomorphan family Reduviidae, which is the best represented group of Heteroptera in terms of rDNA diversity ([47,48], and references therein) suggesting this set of rDNA patterns to be characteristic of true bugs in general.

In most lace bug species (33 from 15 genera of both tribes) where the FISH analysis was performed, the AA pattern is found. It should be noted that in some cases the signals were visible in only one homologue of the bivalent (e.g., in *Agramma minutum*, *Copium brevicorne*, *Acalypta hellenica*, and *Derephysia cristata* from the present study). The same phenomenon or differences in signal strength between the homologues of a bivalent have been repeatedly described in some other families [33,49] and may indicate that ribosomal DNA is a variable region in the true bug genomes with respect to copy number.

The presence of rDNA sites on a pair of autosomes seems to be the most stable evolutionary pattern in lace bugs. This pattern occurs in both Tingini and Acalyptaini, and it probably evolved before the divergence of these tribes. Moreover, the autosomal rDNA position may be the ancestral condition in the family Tingidae as a whole. This pattern seems to predominate in Heteroptera, being found in half of the species studied, and occurs in species differing in chromosome numbers and sex chromosome systems (reviewed in [31]). Specifically, it predominates in the particular families where more data have been accumulated, for example, Reduviidae (e.g., [47,50,51]), Pentatomidae, and Coreidae (e.g., [52,53]). This allows it to be considered as the ancestral condition in Heteroptera as a whole [47,50].

Among Tingini species, two other rDNA patterns, which should therefore be considered as derivative, have been identified in *Agramma femorale*, *A. fallax*, *Dictyla echii*, and *Tingis crispata*, in which 18S rDNA probes hybridized either onto the X-chromosome (in the first species) or onto both X- and Y-chromosomes (in three other species). Note that in each of these genera, the species with the supposed ancestral AA pattern were also present, and each genus showed expressive bias towards this peculiar pattern (Table 2).

The greatest diversity is observed in the genus *Agramma* Stephens, 1829, in which *A. atricapillum* and *A. minutum* retain the ancestral pattern, *A. femorale* displays a single locus on the X-chromosome (X pattern), and *A. fallax* has two loci, one on the X- and the other on the Y-chromosomes (X + Y pattern). This means that the number and distribution of the ribosomal cistrons were undergoing changes during the radiation of *Agramma* despite the fact that all species of this genus kept unchanged the number and size of autosomes and the same XY mechanism of sex determination. A similar situation is observed in the other two lace bug genera, *Dictyla* Stål, 1874, and *Tingis* Fabricius, 1803. In each of these genera, all but one studied species keep the ancestral pattern, whereas *D. echii* and *T. crispata* share the X + Y pattern with *Agramma fallax*, thus supporting its independent origin in each of these three genera. The ability of the rDNA cluster to change its chromosomal position, even among closely related species, has been previously shown for the kissing bug subfamily Triatomini (Reduviidae), which is also homogenous in chromosome number given that almost all species have 20 autosomes [47,50].

How did rDNA loci change their position from autosomal to sex chromosomal between closely related lace bug species? The mechanisms responsible for the shifts remain unknown. However, given that these species retain the unchanged number of autosomes and have an achiasmatic sex chromosome system, those shifts could not be associated with macro-chromosomal rearrangements.

Recent findings indicate that some transposons might be involved in rDNA re-patterning. Some classes of transposons were shown to have the ability to capture entire genes and spread them to different regions of the host genome causing the origin of new loci either followed or not followed by the deletion of the original sites (e.g., [45,48,54,55,56], see also references therein). Transposable elements (known as “jumping genes”) comprise significant proportions of most eukaryotic genomes. The situation observed in lace bugs suggests that the mobility of ribosomal rDNAs during karyotype and species evolution processes could be driven by transposable elements, which are known to be associated with ribosomal DNA loci hence generating changes in their chromosomal distribution [54,57,58]. Note that a similar assumption has been made by many authors who have analyzed rDNA distribution in different groups of animals and plants, e.g., [55,59,60,61,62,63].

A relevant question is whether the rDNA loci tend to occur preferentially in one or another region on the chromosome. Big data analysis (the animal rDNA database is accessible online at www.animalrdnadatabase.com/, accessed on 1 June 2022) shows that rDNA may occur at nearly any chromosomal position, whereas there are significant trends in particular groups, including in some insects [64,65]. For example, differences in 45S rDNA positions seem to exist between two largest and most extensively studied “monocentric” insect groups, Coleoptera and Orthoptera: beetles show a preference for the distal position of 45S rDNA loci, whereas in orthopterans (at least in grasshoppers and crickets), a pericentromeric position is preferred, and terminal locations occur only in exceptional cases [64]. The majority of 45S sites mapped to date in heteropteran species show a terminal localization [31,53]. In lace bugs, on the contrary, rDNA loci seem to show a tendency to localize in the interstitial position ([29], present paper), although in some species they were placed closer to the ends of chromosomes ([26], present paper). Considering the data on ribosomal DNA distribution in heteropteran chromosomes, it is appropriate to take into account that rDNA-FISH analyses in true bugs have almost exclusively been carried out in meiosis, and the compressed structure and small size of meiotic bivalents could prevent the accurate determination of loci positions in many cases.

### 4.4. Lack of the “Insect” Telomere Motif (TTAGG)_n_

In none of the lace bug species analyzed in the present study, FISH revealed hybridization signals of the TTAGG telomeric probe. Taking into account the data received earlier [26,28,29], the absence of the 5 bp motif (TTAGG)_n_, standard and supposedly ancestral for the entire class Insecta [57,66], has been documented to date in 32 species belonging to 15 genera of two tribes of the subfamily Tinginae. Although data are only available for a small percentage of described species (1.2%), they refer to both closely and distantly related lace bug species and support, therefore, the hypothesis that this motif is absent in the family Tingidae in general [26]. It is assumed that the “insect” motif (TTAGG)_n_ was present in a common ancestor of the phylogenetic lineage Cimicomorpha + Pentatomomorpha [67]. However, in further evolution, this motif was repeatedly lost by all families, and the only presently known exception is the family Reduviidae, in which all the species studied so far retain the ancestral motif of telomeres [48,50,67]. The techniques traditionally used to study telomere motifs, both FISH and Southern blot hybridization, detect a specific DNA sequence but, unfortunately, cannot detect a DNA fragment with an unknown DNA sequence [33]. However, as was recently shown by Lukhtanov [68], this problem can be successfully solved based on the analysis of chromosome-level genome assemblies. Currently available genomic data made it possible to identify, in addition to the three previously known short telomere motifs, the 5 bp TTAGG and TCAGG, the 6 bp TTAGGG [66,69,70], 19 new variants of short motifs varying in length from 1 to 17 nucleotides [68]. In the two species of Heteroptera, for which the chromosome-level genome assemblies are available, *Aelia acuminata* (Linnaeus, 1758) (Pentatomidae) and *Acanthosoma haemorrhoidale* (Linnaeus, 1758) (Acanthosomatidae), both from the TTAGG-negative infraorder Pentatomomorpha [33], the deviant 10 bp motifs were TTAGGGATGG and TTAGGGTGGT, respectively [68]. The analysis involving 180 species from 148 genera, 53 families, and 8 orders has shown that the variety of variants of telomere organization in insects is far from limited to the given examples [68]. The applied approach opens up a new very promising avenue to understand how the telomeres are organized and evolve.

## 5. Conclusions

Cytogenetically, the family Tingidae can be characterized by the following main traits: 1. Exclusively stable karyotypes containing in all cases 12 autosomes, which decrease in size more or less linearly; 2. Only two variants of the sex determining system, XY or X(0), the first of which can be considered as evolutionarily ancestral for lace bugs; 3. Four patterns of chromosomal distribution of 18S rDNA loci (on one autosomal bivalent, on both X- and Y-chromosomes, on the X-chromosome, and on one autosomal bivalent + the X), of which the autosomal pattern (AA) is predominant and can be considered as ancestral for lace bugs; 4. Lack of the “insect” telomere motif (TTAGG)_n_, shared with all Cimicimorpha families, except for the family Reduviidae.

## Figures and Tables

**Table 1:** Material used for chromosomal analysis.

Species	Number of Males Examined	Host Plant, Date, and Locality of Collection
Tribe Tingini		
*Agramma blandulum* (Horváth, 1905)	2	*Carex* sp., 1 June 2017, Nizhny Baskunchak vic., Astrakhan Prov., Russia
*A. minutum* Horváth, 1874	7	*Carex* sp., 4 August 2020, Karadag Nature Reserve, Crimea, Russia
*Copium adumbratum* Horváth, 1832	9	*Teucrium* sp., 4 June 2018, Erevan vic., Armenia
*C. brevicorne* (Jakovlev, 1879)	5	*Teucrium* sp., 4 June 2018, Erevan vic., Armenia
*C. clavicorne* (Linnaeus, 1758)	3	*Teucrium* sp., 28 July 2021, Teberda Nature Reserve, Teberda vic., North Caucasus, Russia
*Corythucha arcuata* (Say, 1832)	15	*Quercus* sp., 2 June 2021, Goryachy Klyuch vic., Krasnodar Krai, Russia
*C. ciliata* (Say, 1832)	7	*Platanus orientalis* Linnaeus, 1753, 21 July 2021, Maykop Prov., Republic of Adygea, Russia
*Galeatus affinis* (Herrich-Schaeffer, 1835)	10	*Helichrysum arenarium* Moench, 1794, and *Artemisia marschalliana* Sprengel, 1826, 1–15 July 2021, Voronezh Prov., Russia
*Physatocheilaputshkovi* Golub, 1976	7	*Padus avium*, 27 July 2019, vill. Artybash, Altai Republic, Russia
*Ph.smreczynskii* China, 1952	12	*Padus avium* Linnaeus, 1753, 1–15 July 2021, Voronezh Prov., Russia
*Stephanitisoschanini* Vasiliev, 1935	4	*Malus* sp., 30 May 2018, Khosrov Forest State Reserve, Ararat Prov., Armenia
*Tingis* (*Tingis*) *brevicornis* (Horváth, 1902)	2	Grass community, 30 May 2018, Khosrov Forest State Reserve, Goravan desert, Ararat Prov., Armenia
Tribe Acalyptaini		
*Acalypta gracilis* (Fieber, 1844)	11	Moss community, 20 June 2020, Voronezh Prov., Russia
*A. hellenica* (Reuter, 1888)	2	Moss community, 7 August 2020, Karadag Nature Reserve, Crimea, Russia
*Derephysia.* (*Paraderephysia*) *cristata* (Panzer, 1806)	9	Roots of *Artemisia marschalliana*, 1–15 July 2020, Voronezh Prov., Russia
*Kalama beckeri* (Jakovlev, 1871)	3	Moss community, 7 August 2020, Karadag Nature Reserve, Crimea, Russia

**Table 2:** Cytogenetically studied species of Tingidae.

Species	Meioformula and Pattern of rDNA Localization (in Curly Brackets), References
Tribe Tingini	
1. *Agramma atricapillum* (Spinola, 1837)	12 + XY {AA ^1^}, [29]
2. *A. blandulum* (Horváth, 1905)	12 + XY, present study
3. *A. femorale* Thomson, 1871	12 + XY {X}, [26]
4. *A. fallax* (Horvath, 1906)	12 + XY {X and Y}, [28]
5. *A. nexile* (Drake, 1948)	12 + XY, [22]
6. *A. minutum* Horváth, 1874	12 + XY {AA}, present study
7. *Bredenbachius consanguensis* Distant, 1903	12 + XY, [19]
8. *Cochlochila lewisi* (Scott, 1880)	12 + XY, [20]
9. *Catoplathus carthusianus* (Goeze, 1778)	12 + XY {AA}, [28]
10. *Copium adumbratum* (Horváth, 1891)	12 + XY, present study
11. *C. clavicorne* (Linnaeus, 1758)	12 + XY, [25] 12 + XY {AA}, present study
12. *C. brevicorne* (Jakovlev, 1879)	12 + XY {AA}, present study
13. *C. teucrii* (Host, 1788)	12 + XY {AA}, [28]
14. *Corythucha arcuata* (Say, 1832)	12 + XY {AA}, present study
15. *C. ciliata* (Say, 1832)	12 + XY, [25] 12 + XY {AA}, present study
16. *Dasytingis rudis* Drake and Poor, 1936	12 + XY, [19]
17. *Dictyla echii* (Schrank, 1782)	12 + XY, [25] 12 + XY {X and Y}, [27]
18. *D. humuli* (Fabricius, 1794)	12 + XY, [18] 12 + XY {AA}, [28]
19. *D. platyoma* (Fieber, 1861)	12 + XY {AA}, [29]
20. *D. rotundata* (Herrich-Schaeffer, 1835)	12 + XY {AA}, [27]
21. *Elasmotropis testacea* (Herrich-Schaeffer, 1830)	12 + XY, [25] 12 + XY {AA}, [26]
22. *Galeatus affinis* (Herrich-Schaeffer, 1835)	12 + XY {AA}, present study
23. *G. sinuatus* (Herrich-Schaeffer, 1838)	12 + XY {AA}, [28]
24. *Lasiacantha capucina* (Germar, 1837)	12 + XY, [25] 12 + XY {AA}, [27]
25. *L. hermani* Vasarhelyi 1977	12 + XY {AA}, [29]
26. *Leptobyrsa decora* Drake, 1922	12 + XY, [21]
27. *Melanorhopala clavata* Stål, 1873	12 + XY, [15,16]
28. *Oncochila simplex* (Herrich-Schaeffer, 1830)	12 + XY {AA}, [29]
29. *Physatocheila confinis* (Horváth, 1906)	12 + XY {AA}, [27]
30. *Ph. smreczynskii* China, 1952	12 + XY, [25] 12 + XY {AA}, present study
31. *Ph. putshkovi* Golub, 1976	12 + XY {AA}, present study
32. *Stephanitis caucasica* Kiritshenko, 1939	12 + XY {AA}, [27]
33. *S. nashi* Esaki and Takeya 1931	12 + XY, [17]
34. *S. oberti* (Kolenati, 1856)	12 + XY, [25]
35. *S. pyri* (Fabricius, 1775)	12 + XY {AA}, [27]
36. *S. oschanini* Vasiliev, 1935	12 + XY, present study
37. *S. takeyai* Drake and Maa, 1955	12 + XY, [19]
38. *Teleonemia elata* Drake, 1935	12 + XY, [21]
39. *T. scrupulosa* Stål, 1873	12 + XY, [21]
40. *Tingis* (*Neolasiotropis*) *pilosa* Hummel, 1825	12 + XY {AA}, [29]
41. *T.* (*Tropidocheila*) *caucasica* (Jakovlev, 1880)	12 + XY, [25]
42. *T.* (*Tr.*) *reticulata* Herrich-Schaeffer, 1835	12 + XY {AA}, [29]
43. *T.* (*Tr.*) *sideritis* Štusák, 1973	12 + XY, [25]
44. *T.* (*Tingis*) *ampilata* (Herrich-Schaeffer, 1838)	12 + XY, [18]
45. *T.* (*T.*) *brevicornis* (Horváth, 1902)	12 + XY {AA}, present study
46. *T.* (*T.*) *cardui* (Linnaeus, 1758)	12 + XY, [18] 12 + XY {AA}, [27]
47. *T.* (*T.*) *crispata* (Herrich-Schaeffer, 1838)	12 + XY {X and Y}, [26]
48. *T.* (*T.*) *lasiocera* Matsumura, 1907	12 + XY, [22]
Tribe Acalyptaini	
49. *Acalypta carinata* (Panzer, 1806)	12 + X(0), [25] 12 + X(0) {AA}, [29]
50. *A. gracilis* (Fieber, 1844)	12 + X(0) {AA}, present study
51. *A. hellenica* Reuter, 1888	12 + X(0) {AA}, present study
52. *A. marginata* (Wolff, 1804)	12 + X(0) {AA}, [29]
53. *A. nigrina* (Fallén, 1807)	12 + X(0) [25]
54. *A. parvula* (Fallen, 1807)	10 + XY, [18] 12 + X(0), [25]
55. *Derephysia* (*Paraderephysia*) *longispina* Golub, 1974	12 + X(0) {AA and X}, [29]
56. *D.* (*P.*) *cristata* (Panzer, 1806)	12 + X(0) {AA}, present study
57. *Dictyonota fuliginosa* Costa, 1855	12 + XY, [18]
58. *D. strichnocera* Fieber, 1844	12 + X(0) {AA}, [29]
59. *Kalama beckeri* (Jakovlev, 1871)	12 + X(0) {AA}, present study
60. *K. tricornis* (Schrank, 1801)	12 + X(0), [24,25]

^1^ AA—autosomal bivalent.

## Data Availability

Data supporting reported results can be obtained upon request from the corresponding author (N.V.G.).

## References

[1] Guilbert E., Damgaard J., D’Haese C.A. (2014). Phylogeny of the lacebugs (Insecta: Heteroptera: Tingidae) using morphological and molecular data. Syst. Entomol..

[2] Scudder G.G.E. (1959). The female genitalia of the Heteroptera: Morphology and bearing on classification. Trans. R. Entomol. Soc. Lond..

[3] Štys P., Kerzhner I.M. (1975). The rank and nomenclature of higher taxa in recent Heteroptera. Acta Entomol. Bohemoslov..

[4] Péricart J., Golub V.B., Aukema B., Rieger C. (1996). Superfamily Tingoidea Laporte, 1832. Catalogue of the Heteroptera of the Palaearctic Region II.

[5] Golub V.B., Popov Y.A. (2016). Historical development and problems of classification of the heteropteran insects of the superfamily Tingoidea (Hemiptera: Heteroptera, Cimicomorpha). Meet. Mem. NA Cholodkovsky.

[6] Drake C., Davis N. (1960). The morphology, phylogeny, and higher classification of the family Tingidae, including the description of a new genus and species of the subfamily Vianaidinae (Hemiptera: Heteroptera). Entomol. Am..

[7] Schuh R.T., Štys P. (1991). Phylogenetic analysis of cimicomorphan family relationships (Heteroptera). J. N. Y. Entomol. Soc..

[8] Schuh R.T., Weirauch C. (2020). True Bugs of the World (Hemiptera: Heteroptera). Classification and Natural History.

[9] Schuh R.T., Weirauch C., Wheeler W.C. (2009). Phylogenetic relationships within the Cimicomorpha (Hemiptera: Heteroptera): A total-evidence analysis. Syst. Entomol..

[10] Weirauch C., Schuch R.T., Cassis G., Wheeler W.C. (2019). Revisiting habitat and lifestyle transitions in Heteroptera (Insecta: Hemiptera): Insights from a combined morphological and molecular phylogeny. Cladistics.

[11] Lis B., Zielinska A., Lis J.A. (2022). The King’s Lace Bug *Recaredus rex* Distant, 1909 (Hemiptera: Heteroptera: Tingidae): Systematic Position, First Palaearctic and Afrotropical Records, and Ecological Niche Modelling. Insects.

[12] Lis B. (1999). Phylogeny and classification of Cantacaderini [= Cantacaderidae stat. nov.] (Hemiptera: Tingoidea). Ann. Zool..

[13] Schuh R.T., Cassis G., Guilbert E. (2006). Description of the first recent macropterus species of Vianaidinae (Heteroptera: Tingidae) with comments on the phylogenetic relationships of the family within the Cimicomorpha. J. N. Y. Entomol. Soc..

[14] Golub V.B., Golub N.V. (2019). On the status of the genera complex *Acalypta*, *Dictyonota*, *Kalama* and *Derephysia* (Heteroptera: Tingidae: Tinginae) having common morphological and karyological features. Zoosyst. Ross..

[15] Montgomery T.H. (1901). A study of the chromosomes of the germ cells of Metazoa. Trans. Am. Philos. Soc..

[16] Montgomery T.H. (1906). Chromosomes in the spermatogenesis of the Hemiptera-Heteroptera. Trans. Am. Philos. Soc..

[17] Toshioka S. (1934). On the chromosomes in certain Heterotpera. Oyo-Dobutsugaku-Zasshi.

[18] Southwood T.R.E., Leston D. (1959). Land and Water Bugs of the British Isles.

[19] Jande S.S. (1960). Pre-reductional sex-chromosomes in the family Tingidae (Gymnocerata—Heteroptera). Experientia.

[20] Takenouchi Y., Muramoto N. (1967). A survey of the chromosomes in twenty species of Heteroptera insects. Hokkaido Univ. Educ..

[21] Harley K.L.S., Kassulke R.C. (1971). Tingidae for biological control of *Lantana camara* (Verbenaceae). Entomophaga.

[22] Muramoto N. (1973). A list of the chromosome numbers of Heteropteran insects of Japan. Chromosome Inf. Serv..

[23] Ueshima N. (1979). Animal Cytogenetics. Insecta 6. Hemiptera II: Heteroptera.

[24] Nokkala S., Nokkala C. (1984). Occurrence of the XO sex chromosomes system in *Dictyonota tricornis* (Schr.) (Tingidae, Hemiptera) and its significance for concepts of sex chromosome evolution in Heteroptera. Hereditas.

[25] Grozeva S., Nokkala S. (2001). Chromosome numbers, sex determining systems, and patterns of the C-heterochromatin distribution in 13 species of lace bugs (Heteroptera, Tingidae). Fol. Biol..

[26] Golub N.V., Golub V.B., Kuznetsova V.G. (2015). Variability of 18rDNA loci in four lace bug species (Hemiptera, Tingidae) with the same chromosome number. Comp. Cytogenet..

[27] Golub N.V., Golub V.B., Kuznetsova V.G. (2016). Further evidence for the variability of the 18S rDNA loci in the family Tingidae (Hemiptera, Heteroptera). Comp. Cytogenet..

[28] Golub N.V., Golub V.B., Kuznetsova V.G. (2017). Distribution of the major rDNA loci among four Hemipteran species of the family Tingidae (Heteroptera, Cimicomorpha). Fol. Biol..

[29] Golub N.V., Golub V.B., Kuznetsova V.G. (2018). New data on karyotypes of lace bugs (Tingidae, Cimicomorpha, Hemiptera) with analysis of the 18S rDNA clusters distribution. Comp. Cytogenet..

[30] Grozeva S., Nokkala S. (1996). Chromosomes and their meiotic behavior in two families of the primitive infraorder Dipsocoromorpha (Heteroptera). Hereditas.

[31] Grozeva S., Anokhin B., Kuznetsova V.G., Sharachov I. (2015). Bed bugs (Hemiptera). Protocols for Cytogenetic Mapping of Arthropod Genomes.

[32] Anokhin B., Hemmrich-Stanisak G., Bosch T.C.G. (2010). Karyotyping and single-gene detection using fluorescence in situ hybridization on chromosomes of *Hydra magnipapillata*. Comp. Cytogenet..

[33] Grozeva S., Kuznetsova V.G., Anokhin B.A. (2011). Karyotypes, male meiosis and comparative FISH mapping of 18S ribosomal DNA and telomeric (TTAGG)_n_ repeat in eight species of true bugs (Hemiptera, Heteroptera). Comp. Cytogenet..

[34] Golub N., Anokhin B., Kuznetsova V. (2019). Comparative FISH mapping of ribosomal DNA clusters and TTAGG telomeric sequences to holokinetic chromosomes of eight species of the insect order Psocoptera. Comp. Cytogenet..

[35] Kuznetsova V.G., Grozeva S.M., Nokkala S., Nokkala C. (2011). Cytogenetics of the true bug infraorder Cimicomorpha (Hemiptera, Heteroptera): A review. ZooKeys.

[36] Papeschi A.G., Bressa M.J. (2006). Evolutionary cytogenetics in Heteroptera. J. Biol. Res..

[37] Nokkala S., Nokkala C. (1983). Achiasmatic male meiosis in two species of *Saldula* (Saldidae, Hemiptera). Hereditas.

[38] Nokkala S., Kuznetsova V., Maryańska-Nadachowska A. (2000). Achiasmate segregation of a B-chromosome from the X-chromosome in two species of psyllids (Psylloidea: Homoptera). Genetica.

[39] Nokkala S., Grozeva S., Kuznetsova V., Maryańska-Nadachowska A. (2003). The origin of the achiasmatic XY sex chromosome system in *Cacopsylla peregrine* (Frst.) (Psylloidea, Homoptera). Genetica.

[40] White M.J.D. (1973). Animal Cytology and Evolution.

[41] Grozeva S., Nokkala S., Simov N. (2006). First evidence of sex chromosomes pre-reduction in male meiosis in the Miridae bugs (Heteroptera). Fol. Biol..

[42] Grozeva S., Simov N., Josifov M. (2007). Karyotaxonomy of some European *Macrolophus* species (Heteroptera, Miridae). Mainzer Naturwiss. Arch..

[43] Grozeva S., Kuznetsova V.G., Simov N., Langourov M., Dalakchieva S. (2013). Sex chromosome pre-reduction in male meiosis of *Lethocerus patruelis* (Stal, 1854) (Heteroptera, Belostomatidae) with some notes on the distribution of the species. ZooKeys.

[44] Toscani M.A., Pigozzi M.I., Papeschi A.G., Bressa M.J. (2022). Histone H3 methylation and autosomal vs. sex chromosome segregation during male meiosis in Heteroptera. Front. Ecol. Evol..

[45] Bressa M.J., Papeschi A.G., Vítková M., Kubícková S., Fuková I., Pigozzi M.I., Marec F. (2009). Sex chromosome evolution in cotton stainers of the genus *Dysdercus* (Heteroptera: Pyrrhocoridae). Cytogenet. Genome Res..

[46] Toscani M.A., Pigozzi M.I., Bressa M.J., Papeschi A.G. (2008). Synapsis with and without recombination in the male meiosis of the leaf-footed bug *Holhymenia rubiginosa* (Coreidae, Heteroptera). Genetica.

[47] Bressa M.J., Franco M.J., Toscani M.A., Papeschi A.G. (2008). Heterochromatin heteromorphism in *Holhymenia rubiginosa* (Heteroptera: Coreidae). Eur. J. Entomol..

[48] Panzera F., Pita S., Lorite P., Guarneri A., Lorenzo M. (2021). Chromosome structure and evolution of Triatominae: A review. Triatominae—The Biology of Chagas Disease Vectors. Entomology in Focus.

[49] Pita S., Panzera F., Mora P., Vela J., Palomeque T., Lorite P. (2016). The presence of the ancestral insect telomeric motif in kissing bugs (Triatominae) rules out the hypothesis of its loss in evolutionarily advanced Heteroptera (Cimicomorpha). Comp. Cytogenet..

[50] Poggio M.G., Provecho Y.M., Papeschi A.G., Bressa M.J. (2013). Possible origin of polymorphism for chromosome number in the assassin bug *Zelurus femoralis longispinis* (Reduviidae: Reduviinae). Biol. J. Linn. Soc. Lond..

[51] Bardella V.B., Fernandes T., Vanzela A.L.L. (2013). The conservation of number and location of 18S sites indicates the relative stability of rDNA in species of Pentatomomorpha (Heteroptera). Genome.

[52] Bardella V.B., Fernandes J.A.M., Cabral-de-Mello D.C. (2016). Chromosomal evolutionary dynamics of four multigene families in Coreidae and Pentatomidae (Heteroptera) true bugs. Mol. Genet. Genomics.

[53] Gapon D.A., Kuznetsova V.G., Maryańska-Nadachowska A. (2021). A new species of the genus *Rhaphidosoma* Amyot et Serville, 1843 (Heteroptera, Reduviidae), with data on its chromosome complement. Comp. Cytogenet..

[54] Raskina O., Barber J.C., Nevo E., Belyayev A. (2008). Repetitive DNA and chromosomal rearrangements: Speciation-related events in plant genomes. Cytogenet. Genome Res..

[55] Cabrero J., Camacho J.P.M. (2008). Location and expression of ribosomal RNA genes in grasshoppers: Abundance of silent and cryptic loci. Chromosome Res..

[56] Nakajima R.T., Cabral-de-Mello D.C., Valente G.T., Venere P.C., Martins C. (2012). Evolutionary dynamics of rRNA gene clusters in cichlid fish. BMC Evol. Biol..

[57] Jakubczak J.L., Burke W.D., Eickbush T.H. (1991). Retrotransposable Elements RI and R2 interrupt the rRNA genes of most insects. Proc. Natl. Acad. Sci. USA.

[58] Eickbush T.H., Eickbush D.G. (2007). Finely orchestrated movements: Evolution of the ribosomal RNA genes. Genetics.

[59] Raskina O., Belyayev A., Nevo E. (2004). Activity of the En/Spm-like transposons in meiosis as a base for chromosome repatterning in a small, isolated, peripheral population of *Aegilops speltoides* Tausch. Chromosome Res..

[60] Zhang X., Eickbush M.T., Eickbush T.H. (2008). Role of recombination in the long-term retention of transposable elements in rRNA gene loci. Genetics.

[61] Cabral-de-Mello D.C., Oliveira S.G., de Moura R.C., Martins C. (2011). Chromosomal organization of the 18S and 5S rRNAs and histone H3 genes in Scarabaeinae coleopterans: Insights into the evolutionary dynamics of multigene families and heterochro-matin. BMC Genet..

[62] Proenҫa S.J.R., Serrano A.R.M., Serrano J., Galián J. (2011). Patterns of rDNA chromosomal localization in Palearctic *Cephalota* and *Cylindera* (Coleoptera: Carabidae: Cicindelini) with different numbers of X-chromosomes. Comp. Cytogen..

[63] Symonová R., Majtanova Z., Sember A., Staaks G.B.O., Bohlen J., Freyhof J., Rabova M., Rab P. (2013). Genome differentiation in a species pair of coregonine fishes: An extremely rapid speciation driven by stress-activated retrotransposons mediating exten-sive ribosomal DNA multiplications. BMC Evol. Biol..

[64] Sochorová J., Garcia S., Gálvez F., Symonová R., Kovařík A. (2018). Evolutionary trends in animal ribosomal DNA loci: Introduction to a new online database. Chromosoma.

[65] Sochorová J., Gálvez F., Matyášek R., Garcia S., Kovařík A. (2021). Analyses of the Updated “Animal rDNA Loci Database” with an Emphasis on Its New Features. Int. J. Mol. Sci..

[66] Frydrychová R., Grossmann P., Trubac P., Vitková M., Marec F.E. (2004). Phylogenetic distribution of TTAGG telomeric repeats in insects. Genome.

[67] Grozeva S., Anokhin B.A., Simov N., Kuznetsova V.G. (2019). New evidence for the presence of the telomere motif (TTAGG)_n_ in the family Reduviidae and its absence in the families Nabidae and Miridae (Hemiptera, Cimicomorpha). Comp. Cytogenet..

[68] Lukhtanov V.A. (2022). Diversity and evolution of telomere and subtelomere DNA sequences in insects. bioRxiv.

[69] Kuznetsova V., Grozeva S., Gokhman V. (2020). Telomere structure in insects: A review. J. Zool. Syst. Evol. Res..

[70] Prušáková D., Peska V., Pekár S., Bubeník M., Čížek L., Bezděk A., Frydrychová R.C. (2021). Telomeric DNA sequences in beetle taxa vary with species richness. Sci. Rep..

